# Localized Peptidoglycan Biosynthesis in *Chlamydia trachomatis* Conforms to the Polarized Division and Cell Size Reduction Developmental Models

**DOI:** 10.3389/fmicb.2021.733850

**Published:** 2021-12-09

**Authors:** George W. Liechti

**Affiliations:** Department of Microbiology and Immunology, Uniformed Services University, Bethesda, MD, United States

**Keywords:** cell division, cell size regulation, *Chlamydia*, peptidoglycan, pathoadaptation

## Abstract

Cell size regulation in bacteria is a function of two basic cellular processes: the expansion of the cell envelope and its constriction at spatially defined points at what will eventually become the division plane. In most bacterial species, both cell wall expansion and restriction are dependent on peptidoglycan (PG), a structural polymer comprised of sugars and amino acids that imparts strength and rigidity to bacterial membranes. Pathogenic *Chlamydia* species are unique in that their cell walls contain very little PG, which is restricted almost entirely to the apparent division plane of the microbe’s replicative forms. Very little is known about the degree to which PG affects the size and shape of *C. trachomatis* during its division process, and recent studies suggest the process is initiated *via* a polarized mechanism. We conducted an imaging study to ascertain the dimensions, orientation, and relative density of chlamydial PG throughout the organism’s developmental cycle. Our analysis indicates that PG in replicating *C. trachomatis* can be associated with four, broad structural forms; polar/septal disks, small/thick rings, large rings, and small/thin rings. We found that PG density appeared to be highest in septal disks and small/thick rings, indicating that these structures likely have high PG synthesis to degradation ratios. We also discovered that as *C. trachomatis* progresses through its developmental cycle PG structures, on average, decrease in total volume, indicating that the average cell volume of chlamydial RBs likely decreases over time. When cells infected with *C. trachomatis* are treated with inhibitors of critical components of the microbe’s two distinct PG synthases, we observed drastic differences in the ratio of PG synthesis to degradation, as well as the volume and shape of PG-containing structures. Overall, our results suggest that *C. trachomatis* PG synthases differentially regulate the expansion and contraction of the PG ring during both the expansion and constriction of the microbe’s cell membrane during cell growth and division, respectively.

## Introduction

**Figure 1:**
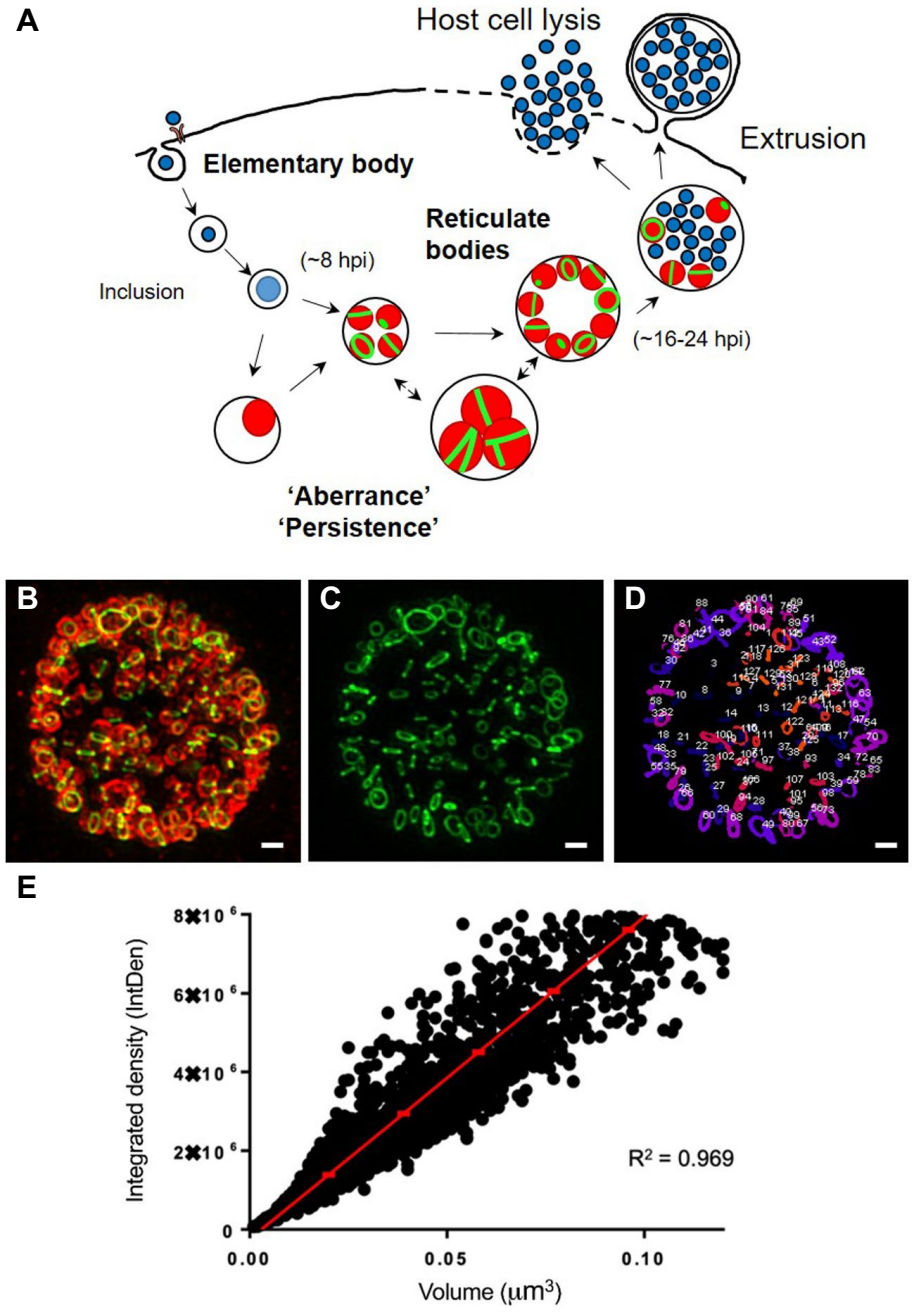
Analysis of peptidoglycan conformation in intracellular *Chlamydia trachomatis*. **(a)** Diagram of the *C. trachomatis* biphasic developmental cycle. Infectious elementary bodies, replicating reticulate bodies, and non-replicating aberrant/persistent forms are shown. **(b–d)** Imaging and analysis workflow for comparing PG-containing structures within chlamydial inclusions. Cell monolayers were infected with *C. trachomatis* L2 434 Bu at an MOI of 1. Infected cells were initially placed on a rocker for 2h and then incubated under static conditions for an additional 22h. At 24 hpi, cells were fixed, permeabilized, and labeled. Z-stacks were collected by Structured Illumination Microscopy (SIM) from fixed cells labeled for PG (green) and the chlamydial Major Outer Membrane protein (MOMP, red; **b**). 3D objects were defined “pixel-by-pixel” from the PG-labeled channel **(c)** utilizing the ImageJ addon 3D Object Counter, enabling each object to be spatially defined. A colorimetric readout was used to compare objects’ relative locations within an inclusion with objects occupying a similar Z plane sharing a common color **(d)**. Scale bars ~1μm. A statistical analysis was subsequently conducted on each individual object within inclusions. Objects were compared by three distinct calculated features: volume, integrated density, and mean fluorescent intensity. **(e)** All PG-containing objects observed within a representative 15 chlamydial inclusions are shown. Object volume and integrated density have been plotted, and the data were observed to fit a linear regression (R^2^=0.969). Object variance from this best-fit regression line corresponds to the relative differences in the mean fluorescent intensities of objects within this data set.

All *Chlamydia* species share a biphasic developmental cycle (Figure 1a). These obligate intracellular pathogens alternate between a small (~0.3μm) extracellular, infectious form (Elementary Body or EB) and a larger (~1μm), intracellular, replicative form (Reticulate Body or RB; Elwell et al., 2016). EB membranes contain a number of highly cross-linked (disulfide bonded), cysteine-rich outer membrane proteins that provide the microbe with the structural rigidity needed for maintaining cell integrity in an extracellular environment. Upon coming into contact with a host cell, EBs are rapidly internalized into a vacuole (termed an “inclusion”) and utilize a type III secretion system (T3SS) to secrete a wide assortment of effector proteins into the cell cytoplasm. Within ~8–12h, EBs begin differentiating into RBs, during which time the outer membrane proteins begin to un-crosslink, the cells enlarge, and begin to replicate. Temporal gene expression largely determines when this transition occurs (Belland et al., 2003), and the expression of early, mid, and late genes roughly corresponds to different phases of the chlamydial developmental cycle (Belland et al., 2003). Sixteen to twenty-four hours after entering a host cell these RBs begin to asynchronously decrease in size (Lee et al., 2018), transition into intermediate forms (IBs), express cysteine-rich outer membrane proteins, and transition into EBs. EBs then exit the cell by one of two routes: (i) the inclusion expands enough to lyse the cell, expelling the newly formed EBs, or (ii) portions of the inclusion begin to “bleb off” into the extracellular environment in a process termed extrusion (Hybiske and Stephens, 2007). The disruption of either of these transition events (EB to RB or RB to EB) effectively halts the developmental cycle and prevents the bacterium from entering new host cells (Beatty et al., 1994).

Peptidoglycan (PG) is an essential structural element in the vast majority of bacterial species (Egan and Vollmer, 2013). This sugar/amino acid polymer encases the bacterium in a mesh-like layer (the sacculus) that provides strength, rigidity, and shape to bacterial cell walls. PG also acts to tether the inner and outer membranes together in Gram-negative bacteria (Egan and Vollmer, 2013) and thus aids in the constriction of the bacterial septum during division (Gray et al., 2015). Nascent PG synthesis is spatially and temporally restricted within bacterial cells *via* two known molecular complexes, each relying on a principle, filament-forming protein: the FtsZ complex (“divisome”), which is associated with septal PG synthesis required during cell division, and the MreB/Rod complex (“elongasome”), which is primarily associated with lateral cell wall synthesis. Members of the Chlamydiae do not encode FtsZ and originally were thought to completely lack PG (Tamura and Manire, 1967; Garrett et al., 1974; Barbour et al., 1982; Fox et al., 1990). Despite the apparent absence of PG, these microbes are still susceptible to PG-targeting antibiotics (Gogolak and Weiss, 1950; Weiss, 1950; Hurst et al., 1953; Moulder et al., 1963; Lin and Moulder, 1966) and this discrepancy was termed the “chlamydial anomaly” (Moulder, 1993). Unlike all other bacterial species described to date, members of the Chlamydiales do not synthesize a PG cell wall or “sacculus” but maintain their PG in a narrow band corresponding to the septal division plane (Liechti et al., 2014, 2016). This PG “ring” plays an active role in *Chlamydia* cell division (Liechti et al., 2016) and is synthesized by a rudimentary MreB/Rod complex. While some *Chlamydia*-like bacteria still utilize a PG sacculus (Pilhofer et al., 2013), evolution within an intracellular environment may have removed the selective pressure to maintain a sacculus for osmotic protection (Mercier et al., 2014; Kawai et al., 2018), thus allowing other members of the Chlamydiales to survive with only enough PG for carrying out the cell division process. As chlamydial PG is immunostimulatory and signals through cytoplasmic NOD receptors (Welter-Stahl et al., 2006), it has been proposed that some *Chlamydia* species have evolved to limit PG utilization, thereby decreasing the pathogen’s immunogenic profile (Liechti et al., 2016; Singh et al., 2020; Brockett and Liechti, 2021).

It has long been held that *Chlamydia* species, like most other bacteria, divide by “classical” binary fission (Gaylord, 1954) in which a parent bacterial cell gives rise to two daughter cells of equal size and volume. However, the very first observations of *Chlamydia* species examined by EM noted that the two membrane-enclosed segments within each RB were either “equal or unequal” in size (Gaylord, 1954). Recent studies suggest that the beginning of the chlamydial division process resembles a polarized mechanism akin to budding, in which a daughter cell initially expands out from one pole of the RB (Abdelrahman et al., 2016; Liechti et al., 2016; Cox et al., 2020). During this initial, polarly restricted expansion of the bacterial membrane, PG localizes to the intermediate zone between the parent and newly expanding daughter cell (Liechti et al., 2016; Cox et al., 2020). A number of earlier studies have reported an initial asymmetry during the division process in a wide array of *Chlamydia* species (Gaylord, 1954; Tanami and Yamada, 1973; Falcieri et al., 1979; Phillips et al., 1984; Hackstadt et al., 1996; Wolf et al., 2000), including some from human endocervical samples (Swanson et al., 1975; Lewis et al., 2014). Despite this initial asymmetry, the chlamydial division process eventually results in two equally sized daughter cells, indicating the presence of an underlying, cell size regulatory mechanism. While “budding-like” division has been described in members of the Planctomycetes (Fuerst, 1995; Lee et al., 2009; Santarella-Mellwig et al., 2013), this process differs significantly from “polarized division” in that it gives rise to daughter cells of different sizes. Polarized division (or “polarized binary fission”) has not been described outside members of the Chlamydiaceae to date, and it is unclear how the process is regulated and what benefit it might confer to pathogenic *Chlamydia* species.

Cell size maintenance in bacteria is principally a function of opposing cellular activities: (i) fatty acid biosynthesis and (ii) the competing processes of localized PG synthesis, remodeling, and degradation. Fatty acids are required for the generation of phospholipids and lipopolysaccharide that compose bacterial cell membranes, whereas PG remodeling is critical for maintaining bacterial cell shape, rigidity and enables the terminal step in the division process (septation). Because pathogenic *Chlamydia* species lack a PG sacculus (Liechti et al., 2014, 2016), but maintain septal PG for cell division and differentiation, the size of a given *Chlamydia* RB should therefore be dependent on the corresponding rates of PG and fatty acid biosynthesis. Most bacteria control cell size utilizing the second messenger (p)ppGpp, which regulates fatty acid biosynthesis (Vadia et al., 2017). Under nutrient starvation conditions, cell size is reduced close to three-fold in most bacterial species (Shi et al., 2021), resulting from the accumulation of ppGpp. By comparison, *Chlamydia* species do not encode the enzymes necessary for ppGpp synthesis (Stephens et al., 1998), and in response to nutrient starvation, RBs frequently increase in size (Harper et al., 2000; Panzetta et al., 2018). When fosfomycin is used to inhibit PG biosynthesis in the *Chlamydia*-related bacterium *Waddlia chondrophila*, bacterial cells similarly continue to increase in size (Jacquier et al., 2014). By comparison, cell division inhibitors that completely abolished PG synthesis in *C. trachomatis* effectively limit cell size expansion (Brockett and Liechti, 2021); however, this is likely due to their effect on global protein synthesis (Bonner et al., 2014; Brinkworth et al., 2018). Another study has reported that *C. trachomatis* RBs undergo cell size reduction prior to differentiating into their non-replicative, infectious forms (Lee et al., 2018), effectively linking the processes of cell size regulation, division, and differentiation. The processes of DNA replication and septation are decoupled in *C. trachomatis* (Lambden et al., 2006); however, the inhibition of replication has been shown to directly affect PG septal formation (Brothwell et al., 2021). The exact mechanism by which RBs regulate their division process and by proxy their cell size, remains unknown.

In this study, we set out to determine how PG synthesis influences the size and shape of chlamydial RBs throughout the pathogen’s division process and developmental cycle. We conducted an analysis of the localization and relative synthesis activity of PG throughout *Chlamydia*’s developmental cycle and compared our data to predictions based on assumptions inherent to both the polarized division model and cell size reduction-preceding-differentiation model of chlamydial development.

## Materials and Methods

### Bacterial Strains and Cell Lines

*Chlamydia trachomatis* serovar L2 strain 434/Bu was provided by Dr. Anthony Maurelli (University of Florida). Chlamydial infections were carried out in HeLa cells or L2 cells (also provided by Dr. Anthony Maurelli) unless otherwise noted. The L2 mouse fibroblast cells and HeLa cell lines were passaged in high glucose Dulbecco’s modified Eagle medium (DMEM, Gibco) and 10% fetal bovine serum (FBS, HyClone). HEK-Blue-hNOD1 and -Null1 cells were purchased from InvivoGen and propagated according to the manufacturer’s instructions.

### Chlamydial Infections

HeLa cells were plated on glass coverslips in 24 well tissue culture treated plate (Costar) at a confluency of ~70–80%. Cells were infected with *C. trachomatis* L2 434/Bu in cold *Dulbecco’s* modified Eagle medium (DMEM; Gibco; 250μl per well) and placed on a rocker in the 37°C tissue culture incubator for 2h. Subsequently, the DMEM was removed and replaced with DMEM supplemented with 10% FBS (HyClone) and 1x MEM Non-Essential Amino Acids Solution (Sigma; 250μl per well). The following aberrance inducing conditions were tested in this study and added at either the time of infection or at 22hpi: 10μM piperacillin, 10μM mecillinam, 10μM penicillin G, 125μM MP265 (polymerization inhibitor of the bacterial cytoskeletal protein MreB), and 3μM chloramphenicol.

### Labeling of Chlamydial Peptidoglycan

HeLa cells were infected with *C. trachomatis* L2 434/Bu at an MOI of 1 unless otherwise stated. Cells and replicating intracellular *Chlamydia* were grown in the presence of the PG intercalating reagent ethynyl-D-alanyl-D-alanine (EDA-DA) at a concentration of 1mM. At 24 hpi, cells were washed 3 times with 1X PBS, permeabilized with methanol and 0.5% Triton X (5min each) prior to blocking with 3% Bovine Serum Albumin (BSA) for 1h and antibody labeling. Click chemistry was carried out to link the PG-associated EDA-DA with conjugated fluorophore Azide-Alexa Fluor 488, as previously described (Liechti et al., 2014, 2016). The chlamydial Major Outer Membrane Protein (MOMP) was labeled with anti-MOMP primary antibody (goat) and secondary antibody (donkey anti-goat Alexa Fluor 594) at the dilutions 1:500 and 1:1,000, respectively. All coverslips were mounted on slides with ProLong Gold Antifade Mounting Media and stored in the dark at 4°C prior to imaging.

### Imaging and Analysis of Peptidoglycan and MOMP-Labeled Objects

All imaging was conducted with a Zeiss ELYRA PS.1 in Structured Illumination (SIM) mode. Settings were fixed at the beginning of image acquisition, Z-stacks were collected for all images taken, and the same parameters were applied for collecting and post-processing all images taken within each experiment. All SIM processing was conducted in RAW fluorescent units (as opposed to averages or percentile standardizations), thus maintaining readouts well below maximum threshold and eliminating the potential for post-processing steps to approach pixel saturation. All post-image processing was conducted on Zen 2012 (Carl Zeiss) software. ImageJ was used for all subsequent image analysis utilizing the FIJI addon “3D Objects Counter” to identify the 3-dimensional spatial constraints of all PG-containing objects within individual *C. trachomatis* inclusions. Relative object volumes were estimated based off of pixel fluorescence data present throughout a Z-stack. Pixels were assigned a “1” or a “0” value based on a common threshold setting applied to all images examined. A composite of all connected “1” pixels through x, y, and z planes was thus constructed from that imaging data. Each PG-labeled object was assigned an estimated volume (in μm^3^), fluorescence mean intensity (average pixel intensity), and integrated density (the sum of all pixel intensity measurements comprising an object). Object size thresholding was restricted to objects larger than 0.001 μm^3^, the detection limit of this approach, corresponding to a single pixel. Statistical significance values were calculated utilizing a one-way ANOVA coupled with a multiple comparisons test with each condition compared with the untreated control group. Inclusion diameters were calculated using the average of the maximum diameter visible from all corresponding z planes and the diameter of a line perpendicular to this maximum diameter.

### Infection of HEK-Blue hNOD1 and Null1 Cells With *Chlamydia trachomatis* and NF-κB Reporter Assay

HEK-Blue cells expressing the hNOD1 receptor and carrying the NF-kB SEAP (secreted embryonic alkaline phosphatase) reporter gene (InvivoGen) were used according to the manufacturer’s instructions and adapted to assess NOD1-specific NF-κB activity during infection with *C. trachomatis*, as previously described (Brockett and Liechti, 2021). Briefly, 3 × 10^5^ cells/mL of HEK-Blue hNOD1 or Null1 cells were plated in 96-well plates (total reaction volume of 200μl per well, ~6.0 × 10^4^ cells per well) and allowed to adhere for 6h at 37°C. These cells were subsequently infected with *C. trachomatis* L2 434/Bu at a MOI of ~1. Plates were centrifuged for 1h at 2,000g to enhance the synchronization of infections and then incubated in a CO_2_ incubator at 37°C. Supernatants were collected at the designated time points for subsequent analysis of SEAP activity. A colorimetric reporter assay was then utilized in order to quantify the abundance of SEAP in cell supernatants. Twenty microliter of supernatants collected from infected cells were added to 180μl of the SEAP-detection solution (InvivoGen) followed by incubation at 37°C for ~8h. SEAP enzymatic activity was then quantified using a plate reader set to 650nm. Infected cells were compared to uninfected controls, as well as infected cells that had been treated with various PG-targeting antibiotics. Uninfected cells treated with the known NOD1 signaling ligand Tri-DAP (1μg/ml) were used as a positive control. All experiments were carried out in parallel in the HEK-Blue Null1 cell line, which contains the empty expression vector but lacks hNOD1. HEK-Blue NOD1 SEAP and Null1 reporter assays were carried out independently in biological triplicate, statistical analysis was conducted by 2-way ANOVA, and significance values were analyzed utilizing Sidak’s multiple comparisons test. Values plotted are means of the raw OD650 measurements.

## Results

### Peptidoglycan Structures Differ in Their Volume and Labeling Intensity Within *Chlamydia trachomatis* Inclusions

To determine the extent to which PG labeling can be utilized to investigate the stages of the *Chlamydia* division process, we carried out a mapping study of all PG-containing objects within 100 chlamydial inclusions. Infected cells were incubated with the PG intercalating agent ethynyl-D-alanyl-D-alanine (EDA-DA; Liechti et al., 2014) and allowed to grow for 24h, at which time the cells were fixed, permeabilized, incubated with cupric sulfate and an azide-conjugated fluorophore in a click chemistry reaction. Cells were subsequently immunolabeled with a monoclonal antibody specific for the pathogen’s Major Outer Membrane Protein (MOMP). Labeled cells were then imaged by structured illumination microscopy (SIM), Z-stacks of infected cell monolayers were collected, all EDA-DA labeled objects within each inclusion were counted and assigned corresponding spatial dimensions and labeling intensities (Figures 1b–d).

We have reported previously that septal (“ring”) PG in *Chlamydia* species is not uniform and can differ in diameter between individual bacterial RBs (Liechti et al., 2016). We have also noted that peptidoglycan in *C. trachomatis* is transient and can dissociate rapidly when the cell division apparatus is inhibited (Liechti et al., 2016) or when “persistence” is induced utilizing various stressors (Brockett and Liechti, 2021). PG labeling intensity is a function of multiple cellular processes: (i) the uptake of the labeling reagent, (ii) the relative rates of its incorporation during peptidoglycan biosynthesis and dissociation during peptidoglycan degradation, and (iii) the relative density of the PG-containing structure. Having observed differences in PG labeling intensities when PG biosynthesis was directly interrupted, we questioned whether PG rings differed significantly in their labeling intensities throughout chlamydial development. We reasoned that if differences exist in either (1) the rates of PG synthesis and PG degradation or (2) the density of PG produced between bacteria at different stages of development, then we should be able to observe corresponding differences in PG labeling intensity. To investigate this question, we plotted PG-containing object volume against object fluorescence integrated density for all labeled objects within 15 chlamydial inclusions (Figure 1e). The overall data fit well within the linear range (*R*^2^=0.969), and we found that as object volume increased, so too did the variance in mean fluorescent intensity labeling, as judged by the distance of each data point relative to the calculated linear regression line for the data set. As integrated density is a function of a labeled object’s volume and mean fluorescent intensity, we reasoned that these differences in the labeling intensities of larger objects could signify differences in either object shape, the density of the labeled PG within objects, or the kinetics of PG assembly and degradation occurring in these structures.

### PG Ring Volume and Labeling Intensities Vary Between RBs at Different Stages of the *Chlamydia* Division Process

**Figure 2:**
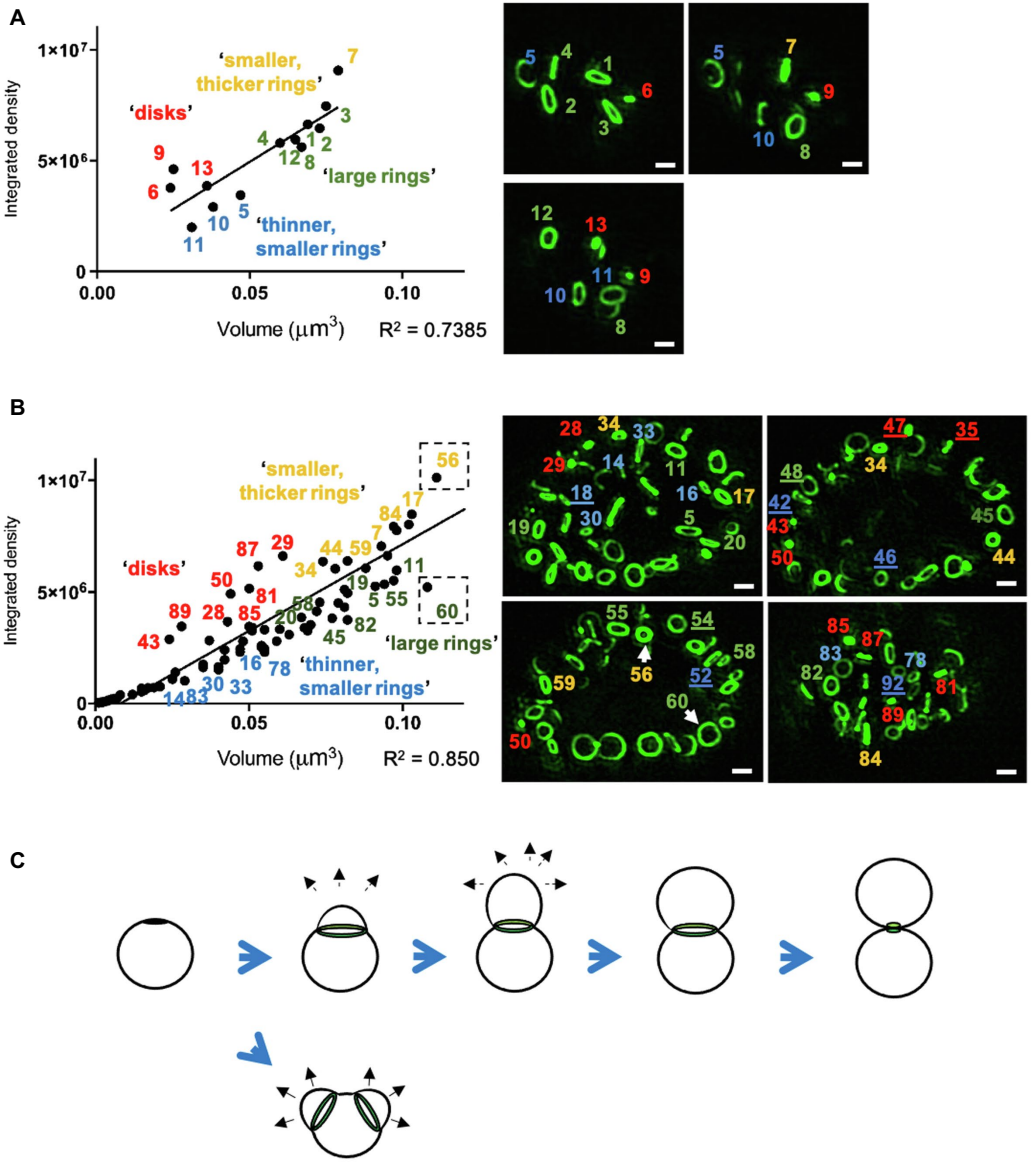
PG ring volume and labeling intensities differ between RBs at distinct stages of the chlamydial division process. **(A,B)** Representative inclusion analysis of all PG-labeled objects within small **(A, < 5μm)** and large **(B, ≥ 5μm)** chlamydial inclusions. Picture panels (right panels, green) represent single imaging planes from two of the 100 independently imaged and analyzed *C. trachomatis* inclusions that were fixed and labeled at 24h post-infection. Image brightness has been increased uniformly across all images so that dimmer objects are more visible. The calculated volumes of the corresponding labeled objects were plotted against each object’s integrated density, which is a function of object volume and mean fluorescent intensity. Distance from the calculated linear regression indicates divergence from the average mean intensity calculated for all objects within an inclusion. PG-labeled objects are numbered, and number color corresponds to the defined group to which each object was assigned. Numerical designations of objects that could not be listed on the data plot are underlined in the image panels. Arrows indicate rings of similar volume but different diameters and labeling intensities. Scale bars ~1μm. **(C)** Illustration of the polarized division model of chlamydial cell division, which is characterized by the heightened density of MOMP labeling and subsequent polar expansion of the cell membrane (black arrows) from one or two locations (as reported by Abdelrahman et al., 2016).

To assess whether the divergence in PG labeling observed in the pooled inclusion data was representative of RB populations within individual inclusions, we repeated our examination for each inclusion from the previous analysis and plotted PG-labeled object volume and intensity measurements for every labeled object within each inclusion. We found similar levels of variance in object mean fluorescent intensity in all inclusions, and examining inclusions containing fewer bacteria allowed us to focus on individual PG-containing objects and compare their relative shapes and staining intensities. For both smaller (<5μm diameter, Figure 2A) and larger (≥5μm, Figure 2B) inclusions, “brighter” objects (high mean fluorescent intensity) appeared to orient as disks or smaller, compact rings (Figures 2A,B; red/yellow), whereas “dimmer” objects (low mean fluorescent intensity) varied in ring diameter and appeared thinner across the ring width (Figures 2A,B; blue/green). Striking differences in labeling intensity between objects of similar volume were found to be the result of relative differences in the apparent thickness of otherwise smaller rings (Figure 2B, lower left panel, objects 56 and 60). Based on these three defining features (shape, volume, and labeling intensity), we found that PG orientation within intracellular chlamydial RBs fit within four broad categories: (i) disks, (ii) small diameter/large volume (≥0.06μm^3^) rings, (iii) large diameter /large volume (≥0.06μm^3^) rings, and (iv) small diameter/small volume (< 0.06μm^3^) rings.

**Figure 3:**
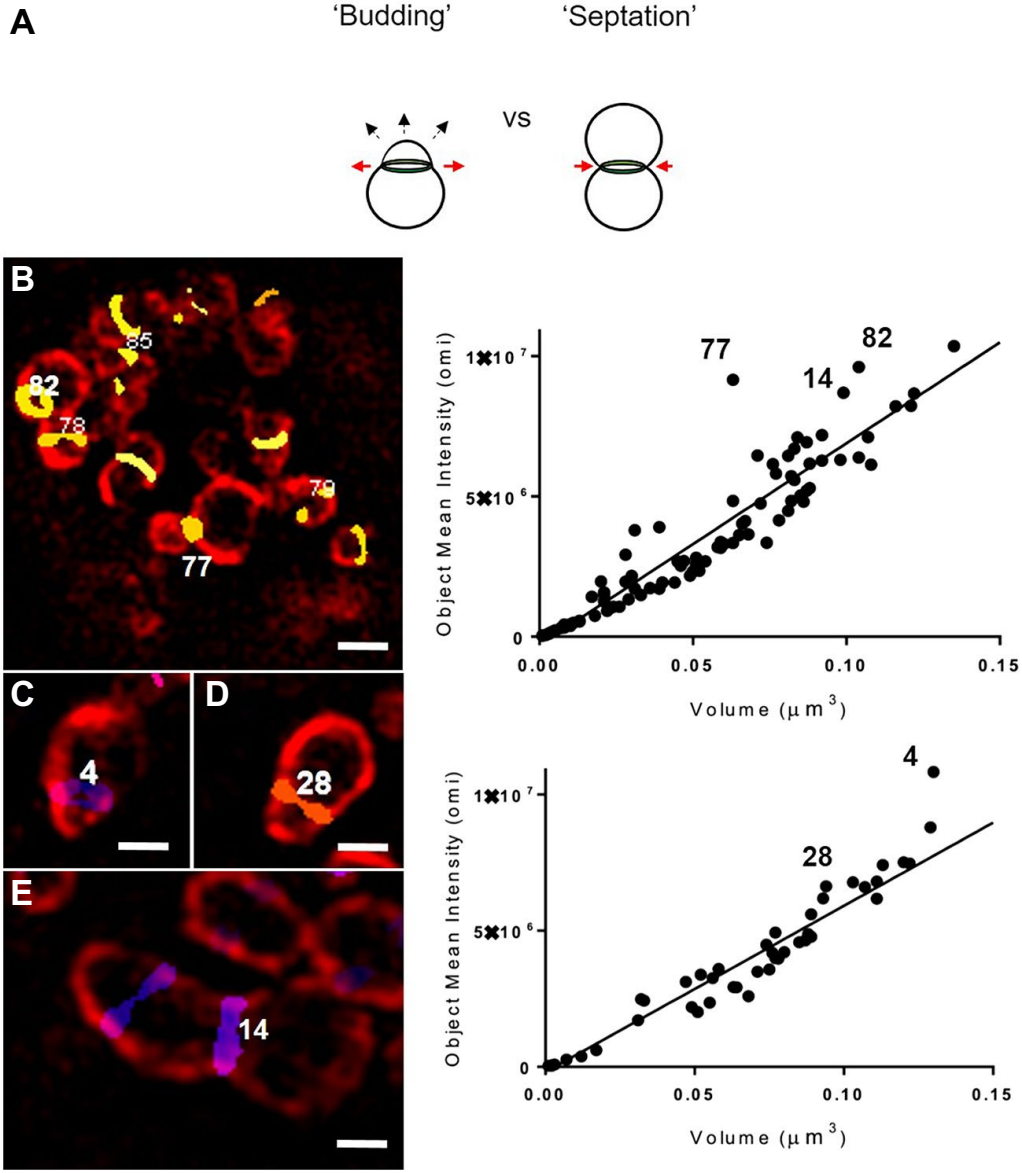
Both “budding” and “septation” processes are associated with PG-labeled objects with higher mean fluorescent intensity values. **(a)** Illustration of the budding and septation stages of the chlamydial division process. The budding phenotype is defined by the polar expansion of the bacterial membrane (dashed arrows) and the expansion of the PG ring (red arrows), whereas septation is defined by the constriction of the PG ring (red arrows), resulting in two daughter cells of equal size. Representative images displaying *C. trachomatis* RBs undergoing either the initial “budding” **(b–d)** or the terminal septation **(e)** stage of their division cycle. RBs are labeled with a monoclonal antibody for MOMP (red) and PG-labeled objects are displayed utilizing a colorimetric “heatmap” corresponding to their relative Z plane positioning within an inclusion. Images are representative of observations made for 51 inclusions >5μm **(b,e)** and 49 inclusions ≤5 um **(c,d)**. Scale bars ~1 μm **(b)** and~0.5 μm **(c–e)**. Each PG-labeled object was assigned a numerical value that can be viewed as a function of its volume and mean intensity measurement (right panels). Variance from the linear regression line represents an object’s relative divergence from the average integrated density for all objects within an inclusion.

We hypothesized that objects with higher-than-average labeling intensities represented PG undergoing rapid synthesis, as would be predicted during septation, the terminal step in bacterial cell division (Figure 2C). However, because it has been reported that *Chlamydia* PG initially localizes to the interface between parent and daughter cell during the initial polar expansion of the cell membrane (Liechti et al., 2016; Cox et al., 2020), we wanted to know whether active PG synthesis was also occurring at this earlier stage of the division process (Figure 3a). We examined PG-labeled objects in 100 labeled inclusions and found that most inclusions larger than 5μm in diameter contained between one to three examples of RBs in which PG was asymmetrically bisecting the bacterial cell, consistent with the “polar budding” phenotype (Figures 3b–d). PG-labeled objects in cells that appeared to be undergoing polar expansion had labeling intensities higher, on average, than all other objects within the inclusions (Figures 3b–d), similar to PG-labeled objects in cells undergoing septation (Figure 3e). Taken together, these data indicate that EDA-DA incorporated PG is maximally present during both the budding and septation phases of chlamydial division.

### PG Ring Volume and Labeling Intensity Is Directly Affected by Protein Synthesis, MreB Polymerization, and Penicillin-Binding Protein Activity

**Figure 4:**
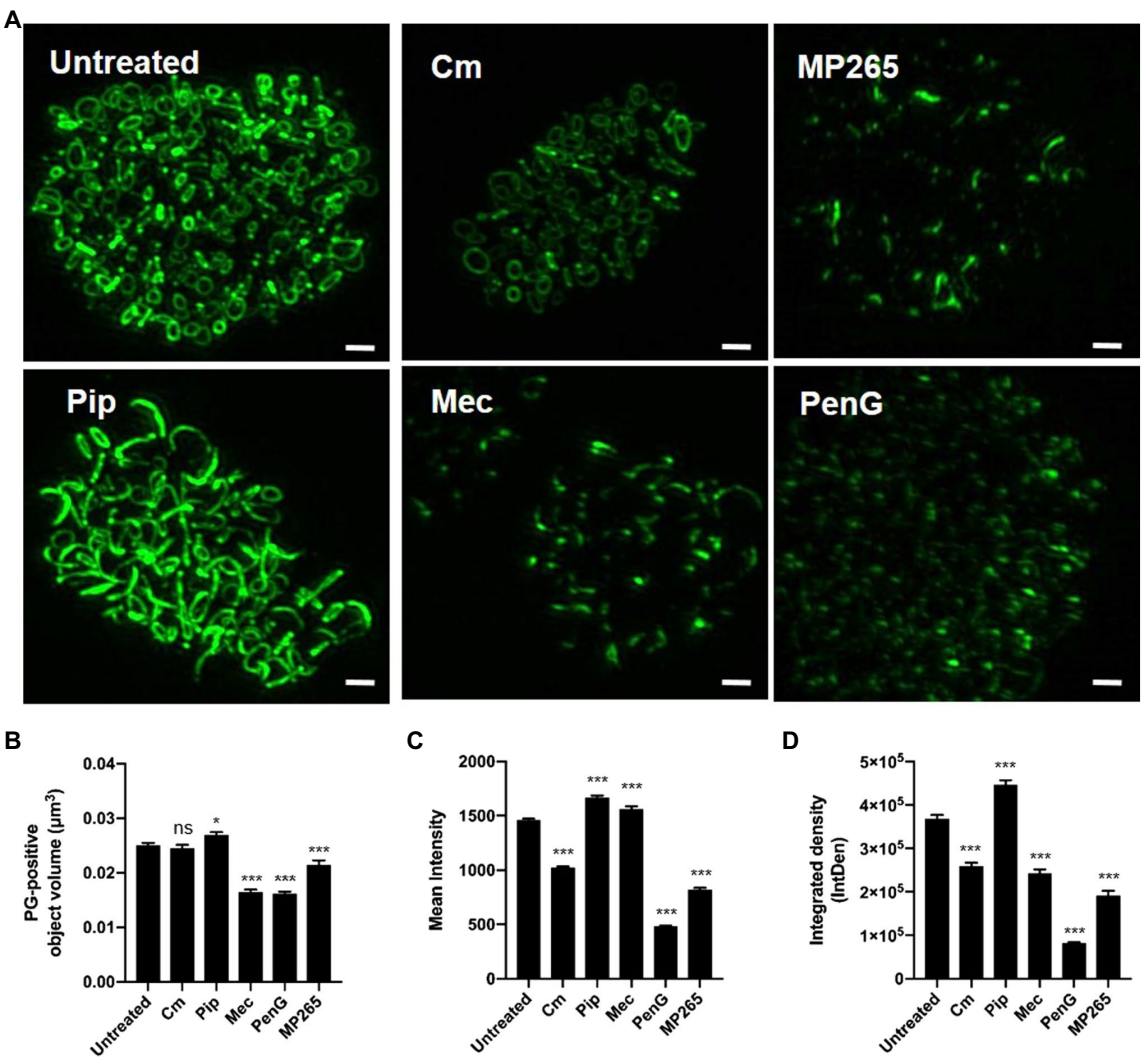
PG ring volume and labeling intensity are directly influenced by protein synthesis, MreB polymerization, and penicillin-binding protein activity. **(A)** PG-labeled objects present within *C. trachomatis* inclusions 24h post-infection that have either been left untreated, or have been treated for 2h with the chemical inhibitors specified. Scale bars ~2μm. Average object volume **(B)**, mean fluorescent intensity **(C)**, and integrated density **(D)** for each group were then compared to untreated controls. A statistical analysis was conducted utilizing one-way ANOVA with multiple comparisons testing. Values represent data acquired from 10 inclusions per experimental group collected over two independent biological replicates. Cm; Chloramphenicol, MP265; MreB polymerization inhibitor, Pip; Piperacillin, Mec; Mecillinam, PenG; Penicillin G. *p<0.05, ***p<0.001, ns; not significant.

Given the differences we observed in labeling intensities for individual PG structures within RB populations, we next wanted to examine how changes to basic molecular processes affected our PG-labeled objects. We hypothesized that object size and shape were dependent on *Chlamydia*’s division cycle and that object labeling intensity, as measured by our assay, is affected by two distinct processes: PG synthesis and degradation. While each of these activities requires protein synthesis, we reasoned that we could delineate which was the prevailing process by incubating cells with a short, sub-inhibitory dose of the protein synthesis inhibitor chloramphenicol (Cm) and measuring the resulting changes in mean object volume and labeling intensity. Cm treatment had no discernable effect on average object volume but did result in decreasing object labeling intensity (Figures 4A–C). By comparison, similar treatment with MP265, an MreB polymerization inhibitor that blocks MreB-dependent PG synthesis in *C. trachomatis* (Liechti et al., 2016), results in large reductions in both PG object volume and labeling intensity (Figures 4B,C). The decrease in mean intensity during Cm treatment suggests that the balance between synthesis and degradation shifts toward turnover when protein synthesis is reduced, however, this degradation appears to be uniform across the entire PG ring as opposed to being localized to specific points on the ring. This may be a result of the timing and kinetics of the assay as previous reports investigating the effects of the inhibition of MreB polymerization on PG in *C. trachomatis* noted an initial reduction in PG ring labeling intensity that was subsequently followed by PG ring degradation (Liechti et al., 2016).

Previous studies investigating the effects of the ß-lactam antibiotic ampicillin on *Chlamydia* ring morphology have demonstrated that while EDA-DA incorporation continues to occur, PG-labeled objects exhibit an enlarged, irregular morphology (Liechti et al., 2014, 2016; Slade et al., 2019; Brockett and Liechti, 2021). This was originally thought to be due to the fact that transpeptidation is not required for the incorporation of EDA-DA, as this process occurs in the cytoplasmic space during Lipid II biosynthesis (Kuru et al., 2019). We wanted to further examine this irregular PG phenotype and establish whether it is unique to the inhibition of specific PBPs in *Chlamydia*. We incubated *Chlamydia*-infected cells for 2h in the presence of inhibitors specific for PBP2 (mecillinam; mec), PBP3 (piperacillin; pip), or both (penicillin G; penG) and compared the relative volume and intensities of PG-positive objects with an untreated control group. When PBP2 activity was inhibited alone, or in conjunction with PBP3, we saw a significant reduction in mean object volume (Figures 4A,B), however, object mean fluorescent intensity did not decrease (Figure 4C). Conversely, inhibition of PBP3 resulted in an increase in object volume (Figure 4A, bottom left panel). The effect was so robust that our subsequent imaging analysis could not distinguish a large number of individual objects, resulting in their removal from the analysis, and a subsequent “low confidence” score in our reported average volume for this treatment condition (Figure 4B). When both PBP2 and PBP3 are inhibited together, both object volume and object labeling intensity was significantly reduced. As an object’s integrated density (IntDen) is a function of both its volume and its labeling intensity, these measurements largely tracked with our mean fluorescent intensity measurements (Figures 4C,D) with the notable exception of the Mecillinam-treated group. Given the relative expansion of the PG ring when PBP3 was inhibited and the relative disassembly of the PG ring when PBP2 was inhibited, our observations suggest that PG structure in *C. trachomatis* is a function of these two differential PG synthases.

**Figure 5:**
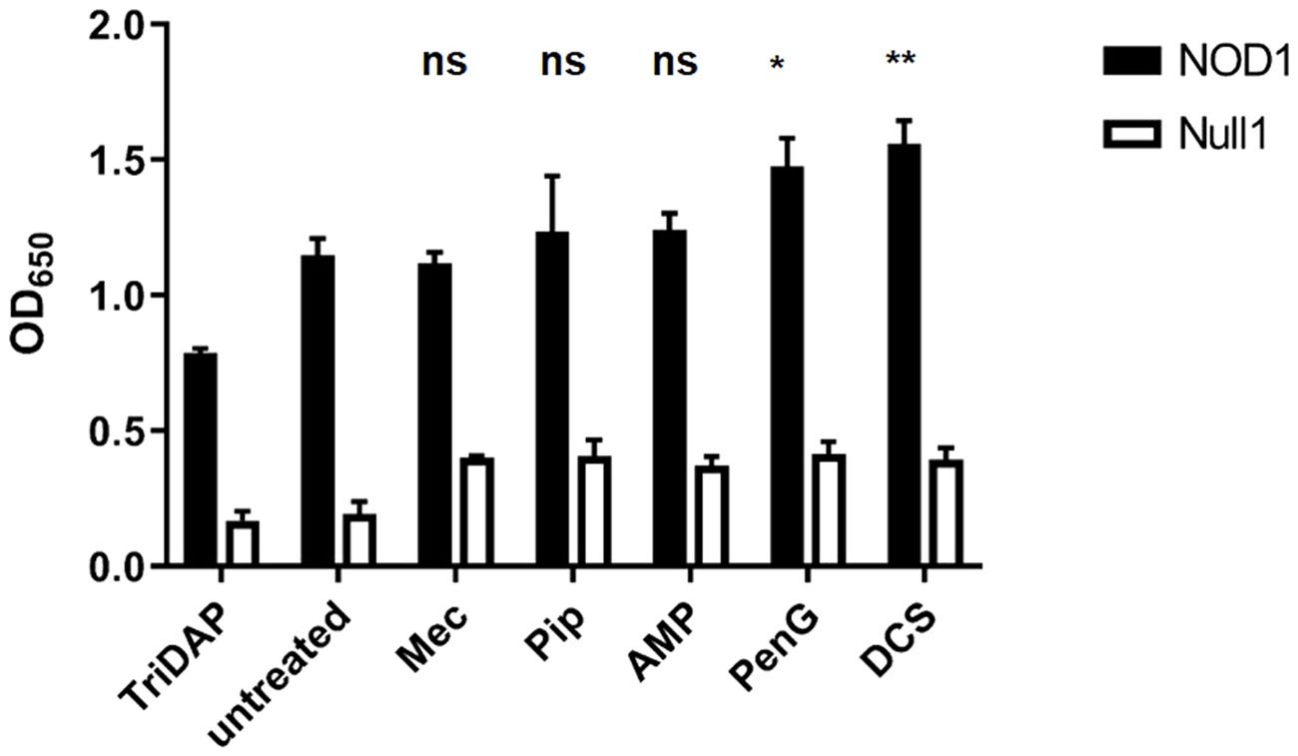
PG synthesis and transpeptidase inhibitors fail to prevent the synthesis of PG precursors in *C. trachomatis*. HEK hNOD1 and HEK Null1 SEAP-reporter cell lines were utilized to quantify the relative abundance of PG-derived muropeptides shed by intracellular *C. trachomatis* in the presence of Mecillinam (Mec), Piperacillin (Pip), Ampicillin (AMP), Penicillin G (PenG), and D-cycloserine (DCS). TriDAP was used as a positive control for NOD1 signaling, and infections were carried out in parallel in a Null1 cell line as a negative control. Data presented are the mean of three independent, biological replicates and error bars represent standard deviation from the mean. A statistical analysis was carried out by 2-way ANOVA with multiple comparisons. Significance values displayed represent comparisons for each group against the untreated control. *p<0.001, **p<0.0001, ns; not significant. No significant difference was observed between all comparisons of the treatments carried out in the Null 1 cell line.

We next wanted to ensure that the loss of PG labeling under our treatment with ß-lactam antibiotics was the result of defects in PG assembly, rather than resulting from the complete inhibition of PG biosynthesis. Gram-negative bacteria are known to continue to synthesize lipid II and attempt to assemble PG despite a block on transpeptidation (Cho et al., 2014). This has previously been demonstrated with D-cycloserine (Packiam et al., 2015) and ampicillin (Brockett and Liechti, 2021) in *C. trachomatis*, utilizing a NOD reporter assay. We examined each treatment condition in a NOD1-signaling reporter cell line that reacts to PG-derived muropeptides produced by *C. trachomatis* that contain meso-diaminopimelic acid (mDAP; Brockett and Liechti, 2021). Similar to ampicillin, we found that *C. trachomatis* continues to generate mDAP-containing muropeptides in the presence of all other ß-lactam antibiotics (Figure 5), indicating that the absence of labeling in our mec and penG treatment conditions is not the result of an absence of Lipid II production. Coupled with our imaging analysis, these data suggest that chlamydial PBP2 and PBP3 activities are associated with different stages of the bacterium’s cell division process. Interestingly, we also found that only the complete interruption of PG assembly (*via* penicillin G) or PG biosynthesis (*via* D-cycloserine) significantly enhanced NOD1-ligand production and release. PG-derived muropeptide turnover was not significantly enhanced when PBP2 or PBP3 functions were selectively inhibited with mecillinam and piperacillin, respectively.

### Average Peptidoglycan Ring Volume and Labeling Intensity Decreases as *Chlamydia trachomatis* Progresses Through Its Developmental Cycle

We next sought to examine PG-labeled objects in the context of the *C. trachomatis* developmental cycle. For this analysis, cell monolayers were infected *via* rocking, as opposed to centrifugation, and infection inoculates were left on the cell layers in order to achieve an asynchronous population of *C. trachomatis* infected cells all at different stages of the microbe’s developmental cycle. Cells were fixed, stained, imaged, and processed as before, but inclusion size measurements were also collected as an indicator of the stage of development that individual inclusions had reached by the point of fixation. *Chlamydia* inclusions fuse and expand throughout the pathogen’s developmental cycle and larger inclusions result from longer incubation times.

**Figure 6:**
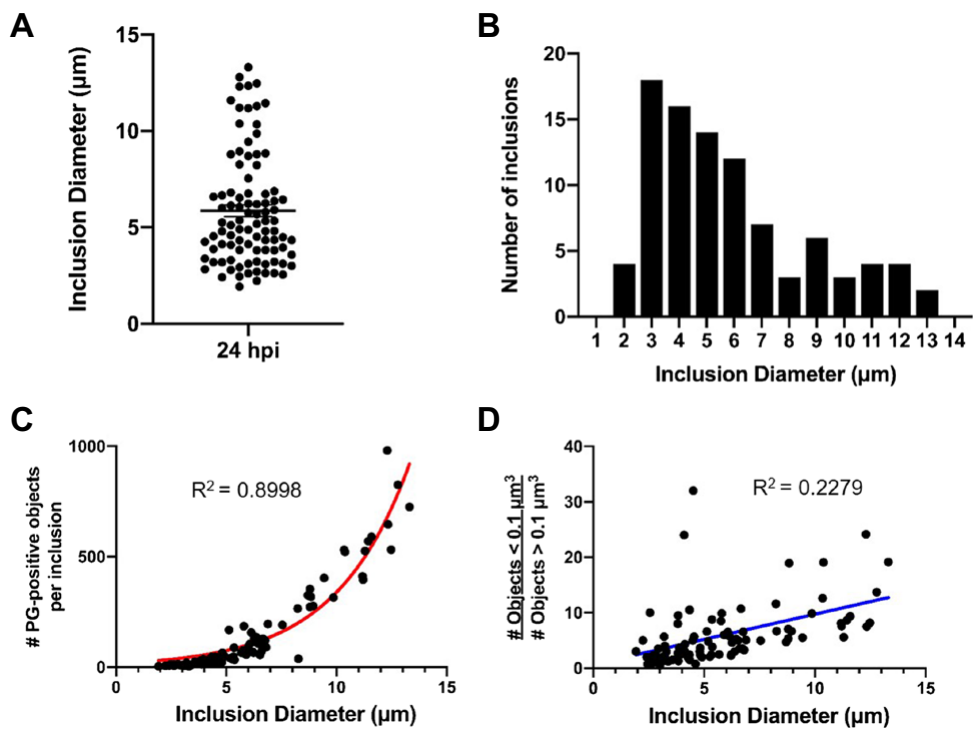
*C. trachomatis* inclusion diameter is an indicator of the relative maturity of the bacterial population that resides within it. Cell monolayers were infected with *C. trachomatis* asynchronously, without centrifugation, as described in Figure 1. **(A,B)** Diameter measurements were taken for 96 chlamydial inclusions that were fixed and imaged 24h post-infection. Data are presented as a point spread from the average inclusion diameter **(A)** as well as *via* histogram **(B)** to show the size distribution throughout the population. **(C)** Inclusion diameter was plotted against the number of PG-positive objects present within each individual inclusion. Data were consistent with a logarithmic growth function (red line). **(D)** The abundance of low confidence objects (> 0.1μm^3^) to high confidence objects (≤ 0.1μm^3^) was plotted as a function of inclusion diameter. Data were inconsistent with a linear regression (blue line).

An analysis of inclusion dimensions revealed a relatively good distribution of inclusion sizes present in our study population (Figure 6A), which exhibited a slight bimodal distribution (Figure 6B). As replicating *Chlamydia* RBs should increase logarithmically over time, we hypothesized that PG-labeled objects should similarly exhibit a logarithmic function when plotted against inclusion diameter. We observed this exact phenomenon (Figure 6C), strengthening our confidence that inclusion diameter could be used as a readout for the relative age of the bacterial population within. In examining PG-labeled objects from individual inclusions, we determined that object volumes ≤0.1μm were of higher confidence than object volumes ≥0.1μm, due to the vast majority of the larger objects resulting from multiple, overlapping smaller objects being mistakenly reported as single large objects. We examined the presence of these larger objects throughout the range of inclusion diameters present in our data set, expecting to find more per inclusion later in development, when the inclusions contain significantly more labeled objects. Surprisingly, we found the reverse to be true, with the ratio of smaller to larger objects increasing as inclusion diameter increased (Figure 6D).

**Figure 7:**
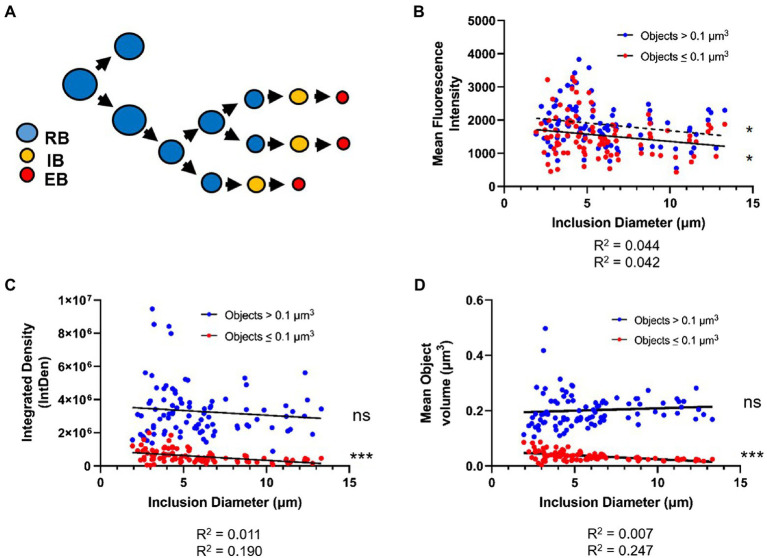
PG-labeled object volume and mean fluorescent intensity decreases as inclusion diameter increases. **(A)** The cell size reduction-preceding-differentiation model, as proposed by Lee et al. The average values for PG-labeled object mean fluorescent intensity **(B)**, integrated density **(C)**, and object volume **(D)** were plotted against inclusion diameter. Objects in each inclusion were separated into those of “low confidence” (>0.1μm^3^) and those of “high confidence” (≤0.1μm^3^). Data for each object type were subjected to a linear regression analysis, and statistical analysis was performed to determine if the linear slope of the data diverged significantly from zero. ns; not significant, *p<0.5, ***p<0.001.

Previous reports have indicated that as *Chlamydia* proceeds through its developmental cycle its replicative forms (on average) decrease in size with subsequent division events (Lee et al., 2018). According to this model, a gradual reduction in RB size eventually enables *C. trachomatis* to drop below a critical size threshold, enabling the pathogen’s replicative form to convert to its infectious form (Figure 7A). This model has been difficult to test utilizing fluorescence microscopy, as delineating individual bacterial cell boundaries within densely packed, intracellular inclusions are challenging. However, given our success mapping PG-labeled objects associated with individual *Chlamydia* RBs, we sought to validate this model by conducting an analysis of the relative volume and density of PG-labeled structures throughout the course of *Chlamydia*’s developmental cycle.

Given the size-reduction-precedes-differentiation model’s constraints, we hypothesized that PG object volume should significantly decrease over time, as a result of *Chlamydia* progressing through its development, while labeling intensity should increase, as it has been proposed that *C. trachomatis* maximizes its replication rate by the mid-stage in its development. We plotted average object volumes, integrated densities, and mean fluorescent intensity values against the diameters of each inclusion for both sets of objects collected (< 0.1μm^3^ and>0.1μm^3^). For objects ≤0.1μm^3^ we found that average object mean fluorescent intensity (Figure 7B), object integrated density (Figure 7C), and object volume (Figure 7D) all decreased as inclusion diameters increased. For objects >0.1μm^3^, no similar decrease in average object integrated density or volume was observed as inclusion diameter increased (Figures 7C,D). Interestingly, objects >0.1μm^3^ did exhibit a similar trend as smaller objects in that their mean fluorescent intensities appeared to significantly decrease as inclusion diameter increased (Figure 7B). Overall, these observations appear to conform to the size-reduction-before-differentiation model in that average PG volume decreases over the span of a developmental cycle.

## Discussion

Cell size regulation is critical for bacterial species, enabling microbes to respond to favorable and detrimental changes in their respective environments. The mechanism(s) employed for regulating cell size are predominantly well-conserved in bacteria; however, the notable exception is members of the Chlamydiales that lack sacculi (Liechti et al., 2014, 2016). For these organisms, cell expansion is presumably limited only by fatty acid biosynthesis and the rate of PG-dependent cell division, rather than the remodeling of a PG-containing cell wall. When PG synthesis is inhibited in these organisms, cell size increases dramatically, indicating that cell size reduction in *Chlamydia* species is largely dependent on the rate of cell division. *C. trachomatis* is capable of halting its biphasic developmental cycle during unfavorable environmental conditions by simply inhibiting PG biosynthesis (Liechti et al., 2016; Slade et al., 2019; Brockett and Liechti, 2021). This prevents cell size-reduction, as well as completely inhibits PG synthesis, enabling the degradation of the PG ring when the pathogen is subjected to iron- and/or tryptophan depletion (Brockett and Liechti, 2021), two conditions utilized by the innate immune system to ward off intracellular bacteria (Feng and Taylor, 1989). These two cell stressors are generally associated with the induction of the pathogen’s aberrant/ persistent state (Figure 1A; Wyrick, 2010).

**Figure 8:**
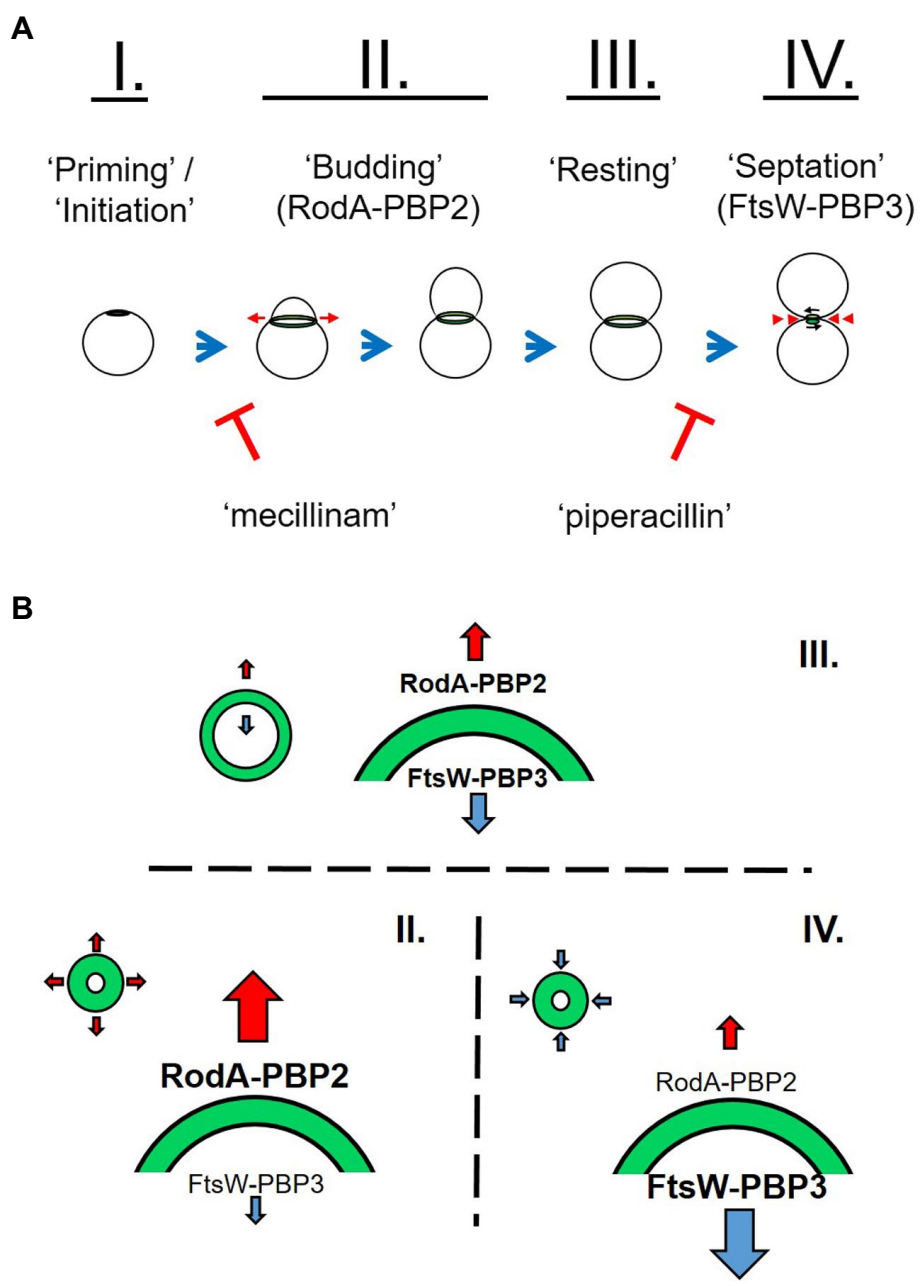
Working model for the role of peptidoglycan in chlamydial polarized cell division. *C. trachomatis* appears to initiate PG synthesis at a single pole **(I.)**. This polar disk expands outward (red arrows) as a result of MreB-dependent RodA-PBP2 PG polymerization on the disk periphery (**A,B**, II.). This coincides with the previously described process of asymmetrical membrane expansion. Due to the transient nature of chlamydial PG in the absence of new synthesis activity, the center of the PG-containing disk degrades, giving rise to a ring-like shape. The ring continues to expand from the outer edge to a point where the rate of PG synthase activity is roughly equivalent to that of PG degradation. This phase of lower intensity PG labeling is the predominant labeling pattern in RBs within *C. trachomatis* inclusions and is indicative of a resting state between the budding and septation stages of the division process **(A,B, III.)**. Upon the initiation of septation, the FtsW-PBP3 PG polymerase acts to synthesize PG on the inner edge of the ring, effectively defining the septum as the ring constricts back into a disk, and ultimately dissociates **(A,B, IV.)**.

While members of the Chlamydiae encode a number of components involved in localized PG synthesis, they lack the central protein responsible for directing the septal division machinery in most other bacterial species; FtsZ (Ouellette et al., 2020). However, these organisms do possess the fundamental elements of the side wall (RodA (Meeske et al., 2016), PBP2) and septal (FtsW (Taguchi et al., 2019), PBP3) PG polymerases. The exact role(s) played by each of these two complexes in the context of i) the newly characterized stages of the *Chlamydia* division process (Figure 8A) and ii) the microbe’s biphasic developmental cycle is only now being actively investigated. Our analysis of the PG composition of 1,000 of chlamydial RBs has led us to conclude that the *Chlamydia* division process can be broken into four stages based on PG localization and intensity measurements: priming/initiation, budding, resting, and septation (Figure 8A). Our observations are consistent with previous reports describing an initial budding stage in the replication cycle of *C. trachomatis* (Abdelrahman et al., 2016; Liechti et al., 2016; Cox et al., 2020), and we found that both the budding and septation phases of the process appear to exhibit the highest rates of PG synthesis, as measured by active incorporation of our labeling probe. These observations suggest that a high degree of PG synthesis activity is characteristic of both the expansion and constriction stages of the PG ring at the initiation and termination of cell division, respectively.

Researchers have hypothesized that *Chlamydia* MreB is capable of directing septal PG biosynthesis in the absence of FtsZ (Ouellette et al., 2012; Liechti et al., 2016), and a recent study has demonstrated that *Chlamydia* MreB is capable of directing septal PG biosynthesis in an *Escherichia coli* strain lacking both MreB and FtsZ activity, effectively enabling this strain to divide in the absence of FtsZ (Ranjit et al., 2020). The ability of the *Chlamydia* MreB protein (MreB^CT^) to effectively direct PG synthesis to the septum in *E. coli* was dependent on the co-expressing of the *Chlamydia* RodZ protein (RodZ^CT^), indicating that these two *Chlamydia* proteins are sufficient to direct septal PG synthesis not only in *C. trachomatis*, but also in surrogate systems. Researchers have previously established that MreB and RodZ are important for directing PG biosynthesis in *C. trachomatis*, and it has been proposed that MreB directs both PG synthases in these organisms (Ouellette et al., 2014; Kemege et al., 2015; Liechti et al., 2016). We found it interesting that despite inhibiting MreB activity, we were still able to observe trace amounts of PG labeling. PG-labeled objects subsequent to MP265 treatment, on average, tended to have smaller volumes and lower mean fluorescent intensities then untreated controls. Previous work investigating the dissociation of the *C. trachomatis* PG ring complex noted that even after dissociation single PG-positive puncta were nearly always observable (Liechti et al., 2016). This suggests that while MreB polymerization is essential for the construction of the chlamydial PG septum, some PG synthesis still occurs in the absence of this process. Alternatively, PG in the vicinity of the divisome complex may simply be spatially protected from degradation by the bacterium’s PG hydrolysis enzymes.

PG biosynthesis and degradation pathways are decoupled in *C. trachomatis*, and inhibition of new synthesis results in the rapid degradation of the PG ring (Liechti et al., 2016). In conducting our analysis of PG-containing structures, we had originally assumed that MreB^CT^ was capable of directing both the RodA-PBP2 and FtsW-PBP3 PG polymerase complexes in *Chlamydia*. Interestingly, we found that when chlamydial PBP2 and PBP3 functions are inhibited separately, significant differences in PG probe incorporation occur (Figure 4). When either MreB or PBP2 function is selectively inhibited, the majority of the PG ring dissociates, however, some PG-labeled foci remain. When accounting for differences in volume (Figure 4C, mean intensity) these foci have comparable levels of PG synthesis activity to untreated and piperacillin-treated controls. These observations are consistent with localized, PG synthesis in the absence of PBP2 activity. By contrast, inhibition of PBP3 results in the accumulation of large PG-containing structures and an aberrant ring morphology similar to that observed upon treatment with ampicillin (Liechti et al., 2014, 2016; Brockett and Liechti, 2021). Interestingly, mean fluorescent intensities of PG-labeled objects in both mecillinam- and piperacillin-treated groups were slightly elevated compared to untreated controls.

When subjected to penicillin and D-cycloserine, bacterial cells often futilely attempt to overcome the perceived defect in their PG synthesis activity by enhancing the production of PG precursor molecules (Cho et al., 2014). As these PG precursors are highly detectable by our innate immune system, this effect can be observed by measuring signaling of NOD1 and NOD2 receptors (Packiam et al., 2015). Utilizing our NOD1 reporter assay, we found that treatment with PBP2- and PBP3-specific inhibitors did not significantly enhance the turnover and shedding of PG-derived muropeptides by *C. trachomatis* while treatment with penicillin G and D-cycloserine did (Figure 5). We took this as evidence that penicillin G is broadly affecting multiple PBPs in *C. trachomatis*, whereas piperacillin and mecillinam appear to be more targeted. D-cycloserine effectively prevents PG biosynthesis at a stage prior to transpeptidation, and therefore, it also significantly enhances NOD stimulation. As our ampicillin treatment group exhibits labeling and immunostimulatory profiles more similar to that of our piperacillin treatment condition, we reason that ampicillin (unlike penicillin G) preferentially inhibits PBP3 in *C. trachomatis*.

Given these differences, we propose that the two chlamydial PG synthases differ in their activities and likely determine PG ring dimensions during cell division (Figure 8B). A previous study found that PG synthesis *via* PBP2 was necessary to initiate polarized division in *C. trachomatis* (Cox et al., 2020), indicating that the PBP2-specific PG ring expansion we characterize in our study is likely essential to this initial division process. We postulate that RodA-PBP2 and FtsW-PBP3 complexes function independently during division and that the differences we observe in labeling localization represent either differences in the location of synthesized PG or that the stability of the PG each synthase generates is inherently different. Given the assumption that PG is a transient structure in *Chlamydia* species, differences in the relative activities of these two synthases would largely explain changes in PG ring dimensions throughout the division cycle (Figure 8B). When combined with our overall assessment of PG localization during the *Chlamydia* division process, we reason that the enhanced labeling we observe during the expansion and constriction of the PG ring is largely due to increased PBP2 and PBP3 activities, respectively.

Our observation that PG ring volume and labeling intensity decrease with time is consistent with the previously proposed cell size-precedes cell differentiation model of *C. trachomatis* development (Lee et al., 2018). Assuming a linear function, we calculate that the average PG-labeled object in an inclusion two microns in diameter (our smallest observed inclusion size) to have an average volume of ~0.047 um^3^, whereas the average PG-labeled object present in an inclusion 13μ in diameter (our largest observed inclusion size) would have an average volume of ~0.016 um^3^. For comparison, Lee et al. found that compared to RB size at 12h post-infection, average RB volume decreased by roughly half by 24hpi and by roughly three quarters by 32hpi (Lee et al., 2018).

Because *C. trachomatis* replicates more rapidly at later developmental stages, it is possible that our observation that PG-labeled objects are smaller later in development is due to the presence of greater numbers of early (budding) and late (septation) division stages with the characteristic smaller PG rings. However, we posit that if there are greater numbers of smaller rings due to bacteria actively synthesizing PG and undergoing budding and septation, then we should have seen corresponding higher relative PG fluorescence mean intensity values, a readout of object density (Figure 2). Because this does not appear to be the case in larger, more mature inclusions (Figure 7B), we conclude that our data support cell size reduction of PG-containing *C. trachomatis* RBs over the span of the pathogen’s developmental cycle. The underlying cause of this decrease in average PG mean intensity throughout the development of *C. trachomatis* is unknown, but may be a function of competing metabolic processes, the availability of some concentration-dependent nutrient, or simply the fact that the essential PG density required for budding/septation differs depending on cell size. Future studies will be needed to further elucidate how cell size affects basic metabolic processes such as PG synthesis and turnover in *Chlamydia* species.

## Data Availability Statement

The raw data supporting the conclusions of this article will be made available by the authors, without undue reservation.

## Author Contributions

GL: conceptualization and design, data curation, formal analysis, investigation, methodology, validation, visualization, writing, editing, funding acquisition, and project administration.

## Funding

This work was supported by a MIRA ESI award (R35 GM138202) and a USU faculty start up award (HP73LIEC18) to GL. The funders had no role in study design, data collection, and interpretation or the decision to submit the work for publication. The opinions and assertions expressed herein are those of the author and do not necessarily reflect the official policy or position of the Uniformed Services University or the Department of Defense.

## Conflict of Interest

The author declares that the research was conducted in the absence of any commercial or financial relationships that could be construed as a potential conflict of interest.

## Publisher’s Note

All claims expressed in this article are solely those of the authors and do not necessarily represent those of their affiliated organizations, or those of the publisher, the editors and the reviewers. Any product that may be evaluated in this article, or claim that may be made by its manufacturer, is not guaranteed or endorsed by the publisher.
